# Diagnosis of Pain Deception Using Minnesota Multiphasic Personality Inventory-2 Based on XGBoost Machine Learning Algorithm: A Single-Blinded Randomized Controlled Trial

**DOI:** 10.3390/medicina60121989

**Published:** 2024-12-02

**Authors:** Hyewon Chung, Kihwan Nam, Subin Lee, Ami Woo, Joongbaek Kim, Eunhye Park, Hosik Moon

**Affiliations:** 1 Department of Anesthesiology and Pain Medicine, College of Medicine, The Catholic University of Korea, Seoul 03312, Republic of Korea; haewon617@gmail.com (H.C.); 1004shelly@naver.com (S.L.); amigo_conmigo@naver.com (A.W.); paparainbow@naver.com (J.K.); hhumil@gmail.com (E.P.); 2 Management of Technology, Korea University, Seoul 02841, Republic of Korea; namkh@korea.ac.kr

**Keywords:** malingering, deception, MMPI, machine learning, psychosocial intervention, pain, logistic model, salivary alpha-amylase, personality tests

## Abstract

*Background and Objectives*: Assessing pain deception is challenging due to its subjective nature. The main goal of this study was to evaluate the diagnostic value of pain deception using machine learning (ML) analysis with the Minnesota Multiphasic Personality Inventory-2 (MMPI-2) scales, considering accuracy, precision, recall, and f1-score as diagnostic parameters. *Materials and Methods*: This study was a single-blinded, randomized controlled trial. Subjects were randomly allocated into a non-deception (ND) group and a deception (D) group. Pain deception, as a form of psychological intervention, was taught to subjects in the D group to deceive the physician. MMPI-2, Waddell’s sign, and salivary alpha-amylase (SAA) were also measured. For analyzing the MMPI-2, the XGBoost ML algorithm was applied. *Results*: Of a total of 96 participants, 50 and 46 were assigned to the ND group and the D group, respectively. In the logistic regression analysis, pain and MMPI-2 did not show diagnostic value. However, in the ML analysis, values of the selected MMPI-2 (sMMPI-2) scales related to pain deception showed an accuracy of 0.724, a precision of 0.692, a recall of 0.692, and an f1-score of 0.692. *Conclusions*: Using MMPI-2 test results, ML can diagnose pain deception better than the conventional logistic regression analysis method by considering different scales and patterns together.

## 1. Introduction

Pain is subjective. It is affected by an individual’s experience or susceptibility. Therefore, the diagnosis of pain itself is made based on a patient’s subjective complaints. Unfortunately, there is still no way to objectively measure pain. Moreover, when a patient deceives a doctor about pain, it is impossible to prove it clearly. As reported by the Diagnostic and Statistical Manual of Mental Disorders, 5th edition (DSM-V), malingering is defined as “the intentional production of false or grossly exaggerated physical or psychological problems motivated by external incentives [1]”. There are not yet a lot of studies on the prevalence of pain deception or malingering. In a 1999 meta-analysis study, the prevalence of pain deception and malingering among chronic pain patients was reported to be between 1.25% and 10.4%, as determined by a pain language questionnaire [2]. In 2009, another study estimated a prevalence of 20~40% depending on the methods used [3]. Accurate diagnosis is important because pain deception is difficult to accurately diagnose due to various diagnostic criteria.

The Minnesota Multiphasic Personality Inventory-2 (MMPI-2) test is a widely used psychological assessment tool that measures various aspects of an individual’s personality, helping to screen for mental health conditions [4]. The diagnosis is made based on a cutoff T-score value of a single scale. However, the validation of such a cutoff value is ambiguous. In addition, it is unclear whether the diagnosis should rely on one or two scales. Therefore, by questioning this, we decided to proceed with a randomized controlled trial (RCT) and analyze the data using machine learning (ML). Although RCT studies have been researched previously in relation to MMPI-2, they have mainly focused on feigned posttraumatic stress disorder (PTSD) [5,6] or fake depression [7], not on pain patients. Therefore, in this study, patients with real low back pain and those without such pain were equally divided between the deception group and the control group. Various methods have been proposed to detect pain deception patients. However, there is no reliable way for clinicians to recognize them [8,9].

In addition, if there is a pain indicator that can distinguish patients from non-patients, it will be easier to identify patients regardless of pain deception. Salivary alpha-amylase (SAA) has been suggested as an indirect biomarker in previous studies [10,11]. Some studies have proposed that there is a correlation between SAA and pain intensity [12,13]. However, Campos et al. [14] and Silva et al. [15] concluded that SAA is not adequate to predict a patient’s pain. Whether SAA is related to pain is still debated, and randomized controlled trials have not been conducted so far.

We hypothesized that pain deception could be detected using MMPI-2 analysis based on an ML algorithm. The primary aim of this study was to evaluate the diagnostic value of pain deception using an ML analysis with MMPI-2 scales. The diagnostic parameters included accuracy, precision, recall, and f1-score. The secondary aim was to evaluate the diagnostic parameters of Waddell’s sign, SAA, and MMPI-2, as defined by the known cut-off value of the T-score.

## 2. Materials and Methods

### 2.1. Study Design

This was a single-blind, parallel, randomized controlled trial. This study was conducted according to the Declaration of Helsinki. It was preregistered at the Clinical Research Information Service (CRIS) of the Republic of Korea (KCT0004876). The Institutional Review Board of The Catholic University of Korea, Yeouido St. Mary’s Hospital approved the study protocol (SC18FNSI0094). All patients provided written informed consent to participate in this study. Participants were enrolled between 21 January 2019 and July 2020.

### 2.2. Participants

The inclusion criteria were as follows: those aged over 20 years; participants who had not experienced pain within one month prior to recruitment (non-patients); patients with chronic low back pain (CLBP) that lasting more than three months, with moderate or severe pain above a numeric rating scale (NRS) of 4/10 (patients); and those who understood this study and voluntarily agreed to participate.

The exclusion criteria were as follows: patients with pain from other diseases; participants diagnosed with sleep disorders or psychiatric disorders, including depression; those who were engaged in litigation or insurance compensation during the screening session; alcoholics and drug addicts; and subjects who could not fill out the MMPI-2, which contained 567 items and took 60 to 90 min to administer by themselves.

### 2.3. Randomization

The participants were randomly assigned into a non-deception (ND) group and a deception (D) group in a 1:1 ratio. The ND group was defined as those who honestly expressed pain, whereas the D group was defined as those who expressed pain excessively to a blinded co-researcher (E.P.). Randomization was stratified according to the existence of CLBP. Randomization numbers, generated using a computer, were placed in sealed envelopes (opaque, not resealable) and opened before the procedure.

### 2.4. Blinding

In this study, only one co-researcher (E.P.) participated in the blind phase, which lasted from random allocation until completion of the MMPI-2 test. The subjects trained in the D group were required to deceive the researcher during the evaluation of pain intensity and Waddell’s sign. The blinded researcher was asked to evaluate the subjects’ facial expressions or responses rather than asking them questions.

### 2.5. Interventions

In this study, pain deception, as a form of psychological intervention, was applied by the D group. All participants were trained for 10 min by a co-researcher on how to respond to a co-researcher during a physical examination and how to complete the NRS, MMPI-2, and SF-MPQ (Short-form McGill Pain Questionnaire). In the D group, participants without pain did deceive a co-researcher (E.P.) into believing they had CLBP and patients with back pain exaggerated their pain as if it was more painful than the actual pain. The degree of pain deception was determined by participants themselves, and they were taught to maintain a consistent level of deception. In the ND group, participants with/without pain were asked to honestly express their pain status to a co-researcher.

### 2.6. Measures

#### 2.6.1. NRS (0, No Pain; 10, the Worst Pain Imaginable)

In this study, two types of NRS were assessed. NRS_real_ refers to the real NRS of participants. It was evaluated by the principal investigator (H.M.). NRS_fake_ refers to the fake NRS of participants in the D group. It was investigated by a blinded co-researcher (E.P.) during the blind phase.

#### 2.6.2. MMPI-2 Scale (Table 1)

MMPI-2 is one of the most commonly used psychological tests. A revision of the original MMPI was published by the University of Minnesota Press. It is a self-report inventory with 567 questions designed to aid in the diagnosis of mental health disorders. The validity of MMPI-2 protocols has been evaluated using the VRIN scale (T-score < 80) or TRIN scale (T-score < 80), with fewer than 30 omitted items [16]. In this study, we focused on scales related to exaggeration, somatic inconvenience, and depression. The following MMPI-2 scales were the main lists examined in this study (a total of 13 scales): exaggeration (F, Fb, Fp), somatic inconvenience (Ds(F-K), KHs, Hy, HEA, Hy4), and depression (D, Ma, RCd, RC2, DEP), defined as sMMPI-2 (selected MMPI-2). Considering that exaggeration, psychological depression, and somatization symptoms are related, these scales were included in this study [17,18]. In this study, exaggeration, somatic inconvenience, and depression using the T-score of MMPI-2 were defined as follows: exaggeration, F or Fb or Fp > 80; somatic inconvenience, either Ds(F-K) or KHs or Hy or HEA or Hy4 > 70; and depression, either D or Ma or RCd or RC2 or DEP > 70 [19,20,21].

#### 2.6.3. Waddell’s Sign

Waddell’s sign is composed of five items: tenderness, simulation, distraction, regional disturbance, and overreaction tests. If more than three items were positive, we assumed that patients had nonorganic pain, particularly low back pain. It is controversial whether Waddell’s sign is related to malingering or not [22]. However, a positive test suggests that a psychological component may be related to a patient’s pain [23].

#### 2.6.4. SF-MPQ

This test was not used for detecting the deception group but for comparing the scores of the two groups. Patients expressed the degree of pain in written sensory and affective words [24]. In this trial, the results of the SF-MPQ provided information on whether the training for the D group was effective by comparing both groups.

#### 2.6.5. SAA

SAA is known to be one of the biomarkers related to psychological stress [25,26]. A correlation between SAA and pain perception has also been suggested [11,14,27,28,29]. Saliva was obtained from each participant using a commercially available cotton sampling device, a Salivary Alpha-Amylase Assay kit (Salimetrics Alpha-Amylase Kinetic/Enzymatic Assay Kit, Salimetrics, CA, USA). SAA was analyzed using an enzyme-linked immunosorbent assay (ELISA), which was performed with a microplate reader (VERSA Max, Molecular Devices, CA, USA). The participants were instructed to rinse their mouths thoroughly with water 10 min before sample collection. The saliva samples were frozen at or below −20 °C within 4 h after sampling. The saliva samples were collected right after group allocating and before educating each group (D and ND groups), as pretending deception could be stressful for participants. All SAA samples were obtained between 0900 h and 1100 h to avoid sampling bias due to the diurnal rhythm [30].

#### 2.6.6. Sample Size

The sample size was calculated based on a study by Dush et al. [31]. The mean values of MMPI-2 in the pain deception and non-deception groups were 67.54 and 21.69, respectively. Based on this effect size, group sizes of 38 accomplished a power of 0.8 to reject the null hypothesis of zero effect size and a significance level of 0.05 using a two-sided z-test. Considering a 30% drop-out rate, 50 patients were deemed appropriate for each group.

#### 2.6.7. Statistical Analysis

The R language version 4.1.3 (R Foundation for Statistical Computing, Vienna, Austria) was used for all statistical analyses. Data are expressed as mean ± standard deviation (SD) for continuous variables, including age, NRS, duration of disease, SF-MPQ score, MMPI-2 score, and SAA value. An unpaired t-test was used to compare mean differences between the D group and the ND group. Data for categorical variables are expressed as the sample number and percentage, *n* (%). A chi-square test was used to determine associations for the categorical variables, such as sex, the presence of CLBP, somatic inconvenience, and Waddell’s sign, between both groups.

Multivariable binary logistic regression analyses were performed to analyze how sMMPI-2 scales and SAA affected the detection of pain deception. The gradient tree boosting (XGBoost) ML algorithm was also applied to predict pain deception. XGBoost is an ensemble learning method consisting of multiple decision trees. To conduct the analysis, two datasets were created with ‘Deception/Non-deception’ as the target variable, distinguishing between the D group and the ND group. The first dataset included 123 predictor variables derived from the whole MMPI-2 (wMMPI-2) dataset, whereas the second dataset was limited to 13 predictors (sMMPI-2) derived from the same dataset. Each dataset was split into 70% training and 30% test sets, and a grid search was conducted to optimize hyperparameters, such as max_depth, learning rate, and subsample, which were then applied to the model. In this case, given the nature of the XGBoost algorithm, which partitions data by splitting the data at specific intervals, we concluded that additional techniques such as k-fold cross-validation would not provide substantial benefits; therefore, we did not implement it. To evaluate the XGBoost ML analyses, accuracy ($\frac{True\;Positive\;\left(TP\right)+True\;Negative\;(TN)}{TP+False\;Negative\;\left(FN\right)+False\;Positive\;\left(FP\right)+TN}$), precision ($\frac{TP}{TP+FP}$), recall ($\frac{TP}{TP+FN}$), and f1-score (2 × $\frac{Precision\times Recall}{Precision+Recall}$) were calculated. The f1-score is a metric corrected for the data imbalance problem that can be overlooked by accuracy. It is suitable for use as a clinical index as it could reduce bias through the weight harmonic average value by reflecting precision and recall. The XGBoost model was developed using the “xgboost” package in R. A *p*-value of < 0.05 was considered statistically significant.

## 3. Results

**Figure 1:**
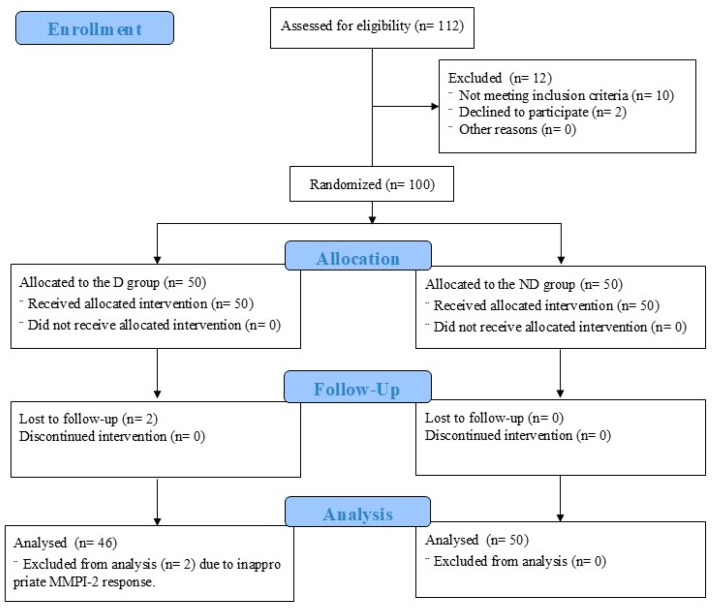
Flow diagram of this study. D, deception; ND, non-deception.

In this study, a total of 100 participants were enrolled (Figure 1). Two participants in the D group were lost to follow-up. Another two participants in the D group were excluded from the analysis due to high TRIN and VRIN results, indicating invalid MMPI-2 responses. Thus, 46 participants in the D group and 50 participants in the ND group were finally analyzed. Both the D and ND groups each consisted of an equal number of normal participants and patients with CLBP.

**Figure 2:**
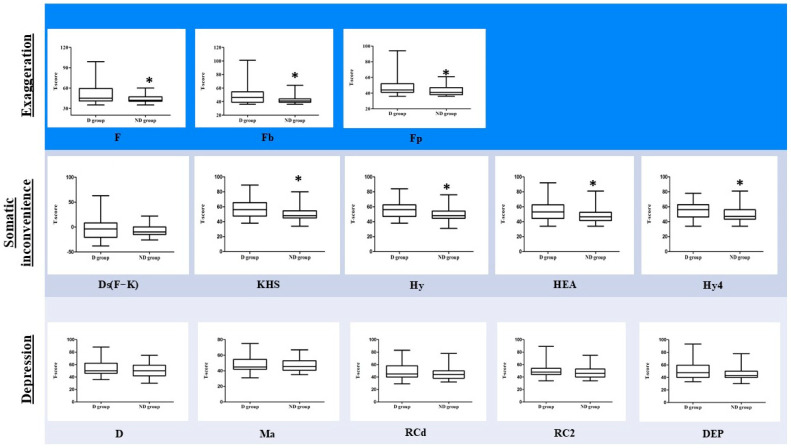
Comparison of sMMPI-2 scales between the D and ND groups. sMMPI-2, selected MMPI-2; D, deception; ND, non-deception. The abbreviations for the MMPI-2 scales are defined in Table 1. sMMPI-2 scales include F, Fb, Fp, Ds(F-K), KHS, Hy, HEA, Hy4, D, Ma, RCd, RC2, and DEP. *: *p* < 0.05.

Table 2 presents descriptive statistics for the subjects in the D group and ND group. Age, sex, duration of disease, and NRS_real_ showed no significant differences between the two groups. Somatic inconvenience, depression, and SAA also did not differ. However, exaggeration (*p* = 0.016), Waddell’s sign (*p* = 0.002), and SF-MPQ (*p* < 0.001) were significantly different between the two groups. T-scores for the D and ND groups were compared, with results shown in Figure 2. Among the sMMPI-2 scales used in this study, all three exaggeration scales (F, Fb, and Fp) and four somatic symptom scales, except Ds(F-K), were significantly different (*p* < 0.05), although all five depression scales showed no significant difference.

**Figure 3:**
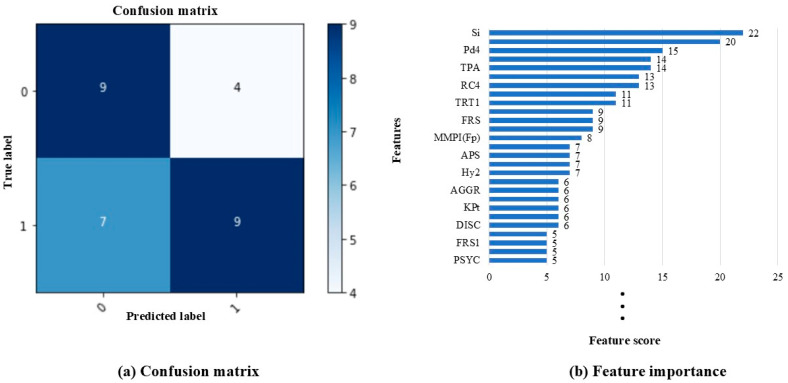
XGBoost analysis of the wMMPI-2 scales to classify the D and ND groups. wMMPI-2, whole MMPI-2; D, deception; ND, non-deception; 0, non-deception; 1, deception. Abbreviations for the MMPI-2 scales are defined in Table 1. (**a**) Confusion matrix; (**b**) feature importance depending on the f1-score.

**Figure 4:**
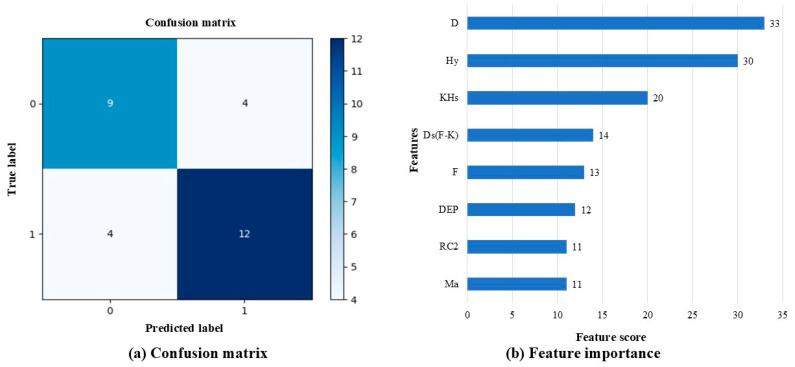
XGBoost analysis of sMMPI-2 scales to classify the D and ND groups. sMMPI-2, selected MMPI-2; D, deception; ND, non-deception; 0, non-deception; 1, deception. Abbreviations for the MMPI-2 scales are defined in Table 1. The sMMPI-2 scales include F, Fb, Fp, Ds(F-K), KHS, Hy, HEA, Hy4, D, Ma, RCd, RC2, and DEP. (**a**) Confusion matrix; (**b**) feature importance depending on f1-score.

The wMMPI-2 and sMMPI-2 scales were analyzed using the XGBoost algorithm to compare the D and ND groups (Figure 3 and Figure 4). In the XGBoost analyses, the sMMPI-2 scale showed a better f1-score compared to the wMMPI-2 scale. No statistically significant difference was observed in the mean comparison between the two groups (Figure 2). However, the inclusion of depression scales in the XGBoost model led to a better improvement in the f1-score. (Table 3).

The diagnostic values of the exaggeration scales (F, Fb, and Fp), somatic inconvenience scales of MMPI-2, and Waddell’s sign for all participants are presented in Table 4. These variables showed good precision values. However, they showed poor accuracy, recall, and f1-scores.

**Figure 5:**
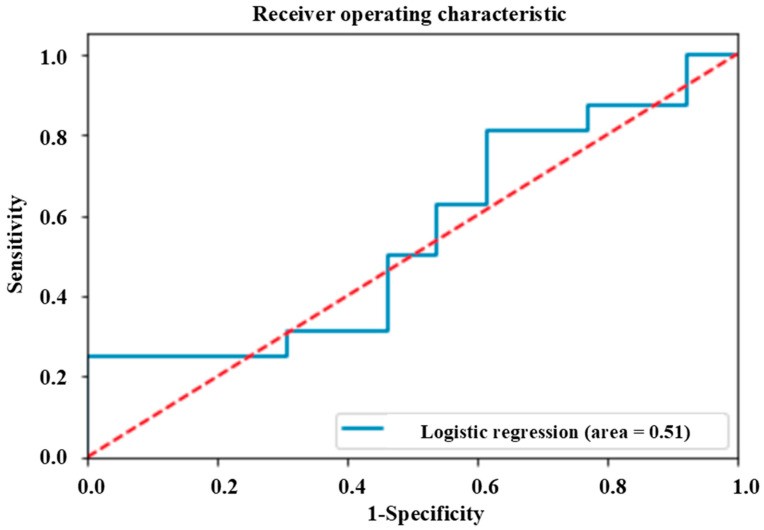
ROC analysis of the logistic regression classifier using sMMPI-2 scales. ROC, receiver operating characteristic; sMMPI-2, selected MMPI-2. The sMMPI-2 scales include F, Fb, Fp, Ds(F-K), KHS, Hy, HEA, Hy4, D, Ma, RCd, RC2, and DEP. Abbreviations for the MMPI-2 scales are defined in Table 1.

There was no relationship between the sMMPI-2 scales and pain deception in the multivariable logistic regression analysis (Table 5). The logistic regression classifier on the test set did not show any diagnostic value for pain deception. The area under the curve (AUC) was 0.51 (Figure 5). The accuracy, precision, recall, and f1-score was 0.52, 0.51, 0.51, and 0.51, respectively.

## 4. Discussion

In this RCT, we aimed to predict pain deception in CLBP using various variables, including MMPI-2. Conventional variables, such as the exaggeration scales (F, Fb, and Fp) of MMPI-2 for pain deception, the somatic inconvenience scales (Ds(F-K), KHs, Hy, HEA, and Hy4) of MMPI-2, and Waddell’s sign, showed good precision values but poor accuracy, recall, and f1-score. However, based on pattern changes in sMMPI-2 scales, including exaggeration, somatic inconvenience, and depression scales, using the ML algorithm, the XGBoost analysis demonstrated better diagnostic value for pain deception than conventional methods, such as logistic regression analysis.

MMPI-2 ML analysis has been used for the diagnosis of various diseases. When MMPI-2 was used for mood disorder screening, its accuracy was 0.87 or higher and its f1-score was between 0.743 and 0.877, depending on the method used [32]. When using ML to predict attention-deficit hyperactivity disorder (ADHD) diagnosis based on the MMPI-2 test, the accuracy was over 90% [33]. These studies used ML because of its excellent reliability and scalability in terms of prediction. In this study, conventional analysis using the exaggeration scales or somatic inconvenience scales of MMPI-2 or Waddell’s sign showed a high precision value despite low recall and f1-score values, with f1-scores below 0.4. Although the results of conventional analysis for diagnosing pain deception can provide a basis for a definite diagnosis, they have poor diagnostic value for use in screening tests. For screening purposes, a higher f1-score, which reflects a better balance between precision and recall, is essential for meaningful predictions. In this research, the ML analysis of MMPI-2 demonstrated a significant improvement in f1-scores, suggesting the potential for balanced predictions, even though its precision values were lower compared to conventional methods. This result suggests that ML analysis can provide a high-performance classification model as a screening tool for pain deception, rather than a confirmed diagnosis.

Pace et al. investigated the diagnostic value of b-Test error scores for detecting malingerers among cognitively impaired patients using ML techniques and leave-one-out cross validation (LOOCV) [34]. This LOOCV analysis reported an f1-score of 0.9. Although this f1-score is high, it is important to note that LOOCV analysis primarily serves as a validation technique to assess a model’s generalization performance. In contrast, although the current study’s f1-score is lower, the XGBoost analysis is designed for predictive tasks and may offer a more suitable approach for identifying malingering groups, as it focuses on optimizing prediction accuracy rather than generalization.

The F, Fp, and Fb scales, commonly used to identify pain deception or exaggeration of psychological problems in MMPI-2, were found to be insignificant in the regression analysis in our study. Despite the application of ML methods, these scales did not rank in the top three. In contrast, although the depression scales showed no statistical significance in the t-test, they contributed to improved model performance in the XGBoost analysis by interacting with other variables, ultimately placing in the top three. Unlike regression analysis, which focuses on individual scales, tree-based models such as XGBoost analysis can leverage even weak signals from multiple scales across numerous trees, enhancing both model complexity and performance.

In addition, variables representing somatic inconvenience, such as Hy and KHs, also showed a high rank, suggesting that they could be used in addition to the f1-score to detect pain deception. This result partially supports a previous study suggesting that elevated Hy, Hs, and FBS scales might indicate somatic pain deception [35]. The Hy scale is related to psychiatric symptoms, such as paranoia and delusions, and the KHs scale assesses excessive worry about health, often involving exaggerated concern over minor or non-existent symptoms. The XGBoost analysis can capture complex patterns and non-linear relationships, allowing even non-significant features in the t-test to contribute positively to prediction. When interpreting MMPI-2 test results, various scales and patterns should be considered together. It is inappropriate to interpret results based on only one scale. Therefore, it is necessary to comprehensively assess the results of the MMPI-2 test and judge whether they are related to pain deception.

This study has a few limitations. First, due to the nature of psychological interventions, it was difficult to confirm how well participants understood and adhered to the experiment, especially for the MMPI-2 test, which required a significant amount of time and posed numerous questions. Therefore, it was unclear whether participants maintained a simulated state over an extended period, despite receiving adequate education beforehand and having no secondary gain. Second, this study did not accurately determine the cut-off value for diagnosing pain deception. Lastly, the data volume may have been insufficient for conducting the ML analysis, particularly given the small sample size, which could affect the model’s robustness. Additionally, we did not evaluate the model’s performance across different data splits or datasets, which limits our ability to assess its consistency. Although ML algorithms are designed to maximize clinical significance, a large sample size may yield different results. Future studies should focus on actual participants with secondary gain. Also, it is essential to collect more data and apply methods such as fMRI, EEG, and pain biomarkers to objectively validate the results.

## 5. Conclusions

In conclusion, the results of this study suggest that diagnosing pain deception through pattern changes in MMPI-2 scales using ML could be valuable. This approach may benefit clinicians to by enabling more exact and objective detection of deception in various situations. Further large-scale studies are needed to screen and predict pain deception more precisely.

## Figures and Tables

**Table 1:** List of abbreviations of MMPI-2 scales used in this study.

Abbreviation	Scale Composition	Description
VRIN	Validity indicators	Variable response inconsistency
TRIN	Validity indicators	True response inconsistency
F	Validity indicators	Infrequency
Fb	Validity indicators	F back
Fp	Validity indicators	F-psychopathology
K	Validity indicators	Correction
Ds(F-K)	Validity indicators	F minus K
KHs	Clinical scales	Hypochondriasis
Hy	Clinical scales	Hysteria
HEA	Content scales	Health concerns
Hy4	Clinical subscales	Complaining of physical symptoms
D	Clinical scales	Depression
Ma	Clinical scales	Hypomania
RCd	Restructured clinical scales	Demoralization
RC2	Restructured clinical scales	Low positive emotion
DEP	Clinical scales	Depression
Si	Clinical scales	Social introversion
Ho	Supplemental scales	Hostility
Pd4	Clinical subscales	Social deviate

**Table 2:** Descriptive statistics according to subject group.

	D Group (n = 46)	ND Group (n = 50)	*p*-Value
Age, yr	44.3 ± 14.9	48.1 ± 13.8	0.205
Sex, M/F	26/20	25/25	0.527
Low back pain, Y/N	23/23	25/25	1.000
Duration of disease, months	8.7 ± 16.5	7.1 ± 19.1	0.672
NRS_real_	2.5 ± 2.6	2.4 ± 2.5	0.851
NRS_fake_	6.9 ± 2.1	2.4 ± 2.6	<0.001
Exaggeration, Y/N	5/41	0/50	0.016
Somatic inconvenience, Y/N	8/38	4/46	0.168
Depression, Y/N	15/31	10/40	0.163
Waddell’s sign, Y/N	12/34	2/48	0.002
SF-MPQ	36.9 ± 13.4	12.5 ± 11.7	<0.001
SAA, U/mL	123.3 ± 181.1	125.6 ± 98.5	0.940

D, deception; ND, non-deception; M/F, male/female; NRS_real_, the real numeric rating scale; NRS_fake_, the fake numeric rating scale; SF-MPQ, Short-form McGill Pain Questionnaire; SAA, salivary alpha-amylase.

**Table 3:** XGBoost analysis of the MMPI-2 scales to classify the D and ND groups.

	Accuracy	Precision	Recall	f1-Score	Top three Features
wMMPI-2 scales	0.621	0.692	0.562	0.651	Si, Ho, Pd4
sMMPI-2 scales	0.724	0.692	0.692	0.692	D, Hy, KHs
wMMPI-2 without depression scales	0.586	0.500	0.667	0.571	Ho, Si, Rc4
sMMPI-2 without depression scales	0.552	0.625	0.588	0.606	Fb, Fp, Ds(F-K)

D, deception; ND, non-deception; wMMPI-2, whole MMPI-2 scales; sMMPI-2, selected MMPI-2 scales, including F, Fb, Fp, Ds(F-K), KHS, Hy, HEA, Hy4, D, Ma, RCd, RC2, and DEP. Abbreviations for the MMPI-2 scales are defined in Table 1.

**Table 4:** Diagnostic values of the exaggeration scales and somatic inconvenience scales of MMPI-2 and Waddell’s sign.

Variables	Accuracy	Precision	Recall	f1-Score
Exaggeration scales of MMPI-2	0.573	1.000	0.109	0.196
Somatic inconvenience scales of MMPI-2	0.563	0.667	0.174	0.276
Waddell’s sign	0.625	0.857	0.261	0.400

Exaggeration scales of the MMPI-2 scales: F, Fb, and Fp; somatic inconvenience scales of MMPI-2: Ds(F-K), KHs, Hy, HEA, and Hy4. Abbreviations for the MMPI-2 scales are shown in Table 1.

**Table 5:** Multivariable logistic regression analysis of the relationship between the sMMPI-2 scales and pain deception.

MMPI-2 Scales	OR	95% CI		*p*-Value
F	1.06	−0.10	0.22	0.477
Fb	0.99	−0.19	0.18	0.945
Fp	1.05	−0.09	0.18	0.492
Ds(F-K)	1.08	−0.01	0.17	0.072
KHs	1.11	−0.10	0.32	0.319
Hy	1.13	−0.10	0.26	0.076
HEA	0.83	−0.38	0.02	0.076
Hy4	1.01	−0.13	0.16	0.854
D	0.93	−0.20	0.04	0.199
Ma	0.93	−0.16	0.02	0.131
RCd	0.94	−0.26	0.12	0.485
RC2	1.02	−0.01	0.12	0.667
DEP	1.03	−0.16	0.22	0.755

sMMPI-2, selected MMPI-2 scales, including F, Fb, Fp, Ds(F-K), KHs, Hy, HEA, Hy4, D, Ma, RCd, RC2, and DEP; OR, odds ratio; CI, confidence interval. Abbreviations for the MMPI-2 scales are shown in Table 1.

## Data Availability

The datasets used and/or analyzed in the current study are available from the corresponding author upon reasonable request.
